# Coordinated regulation of propionyl-CoA carboxylase subunits drives precursor flux optimization in spinosad production

**DOI:** 10.3389/fmicb.2025.1643527

**Published:** 2025-09-16

**Authors:** Ziyuan Xia, Xiaomin Li, Xirong Liu, Li Cao, Duo Jin, Zirui Dai, Baolong Bai, Qian Liu, Jie Rang, Zirong Zhu, Liqiu Xia

**Affiliations:** ^1^Hunan Provincial Key Laboratory for Microbial Molecular Biology, State Key Laboratory of Developmental Biology of Freshwater Fish, College of Life Science, Hunan Normal University, Changsha, Hunan, China; ^2^Hunan Norchem Pharmaceutical Co., Ltd., Changsha, Hunan, China

**Keywords:** *Saccharopolyspora spinosa*, spinosad, propionyl-CoA carboxylase, alanine, compensatory regulation

## Abstract

Spinosad, a natural agricultural pesticide produced by *Saccharopolyspora spinosa*, has gained global attention owing to its potent insecticidal properties and environmental compatibility. To enhance spinosad biosynthesis, the propionyl-CoA carboxylase (PCC) complex responsible for precursor synthesis was investigated in this study. Six engineered strains were constructed using clustered regularly interspaced short palindromic repeats interference (CRISPRi) and overexpression strategies to systematically regulate the core PCC subunits encoded by pccA, pccB1, and pccB2. Upregulation of these genes enhanced PCC activity, improved energy and precursor utilization, and activated metabolic pathways, ultimately leading to increased spinosad production. Contrary to expectations, CRISPRi-mediated suppression of pccB1 resulted in the highest-yielding strain, demonstrating a 2.6-fold increase in production over the wild type. Integrated quantitative reverse transcription-polymerase chain reaction, heterologous expression, western blot, and proteomic analyses revealed compensatory regulation within the PCC family. pccB1 downregulation significantly upregulated pccA and pccB2 expression while enhancing PCC enzymatic activity. Subsequent proteome-guided supplementation of amino acids critical for spinosad precursor synthesis augmented the yields. The optimized *S. spinosa*-pSET-dCas9-pccB1 strain, supplemented with alanine, achieved a remarkable 6.2-fold increase in production compared with the parental strain, establishing an effective strategy for metabolic engineering of spinosad biosynthesis.

## 1 Introduction

Spinosyns, a family of macrolide secondary metabolites biosynthesized by *Saccharopolyspora spinosa* (CCTCC M206084), are recognized as environmentally benign biopesticides owing to their broad-spectrum insecticidal efficacy, low environmental persistence, and minimal residual impact (Malhat and Abdallah, 2019). The characteristic structure of spinosad comprises a 21-membered polyketide-derived macrocyclic lactone featuring a unique tetracyclic core system conjugated with two deoxysugars: D-forosamine and tri-*O*-methyl-L-rhamnose. This complex architecture is assembled via a coordinated polyketide synthase (PKS) system consisting of five modular enzymes (SpnA–E) that utilize acetyl-CoA, propionyl-CoA, malonyl-CoA, and methylmalonyl-CoA as essential precursors (Sparks et al., 2020).

Despite its substantial commercial potential as an ecofriendly agricultural agent, industrial-scale production of spinosad remains constrained by suboptimal fermentation yields (Tao et al., 2018). Current metabolic engineering strategies, such as precursor flux optimization and regulatory pathway manipulation, have achieved limited success in addressing the fundamental biosynthetic limitations. This production bottleneck continues to hinder the global supply–demand balance, necessitating innovative approaches in synthetic biology and pathway engineering to overcome the inherent metabolic constraints of the native producer.

Advancements in analytical technologies for metabolomics have enabled the systematic enhancement of natural product biosynthesis via strategic metabolic engineering (Li et al., 2021). Three synergistic approaches have been proposed to optimize precursor availability: (1) amplification of bottleneck enzyme-coding genes to overcome rate-limiting steps (Chen et al., 2020), (2) suppression of competitive pathway fluxes to minimize metabolic crosstalk, and (3) dynamic redistribution of primary metabolic resources toward target biosynthetic pathways (Krysenko and Wohlleben, 2024). Complementarily, overexpression of global regulatory factors, such as TetR-family transcriptional activators, has proven effective in upregulating secondary metabolite gene clusters (Yu et al., 2025).

Successful cases have demonstrated the universality of precursor-centric strategies. In *Streptomyces diastatochromogenes*, guanosine triphosphate pool expansion considerably improved toyocamycin biosynthesis (Zhang et al., 2022). Similarly, *Streptomyces avermitilis* engineered for elevated acyl-CoA precursor levels achieved a 2.3-fold increase in avermectin B1a titers (Hao et al., 2022). These findings establish that precursor flux modulation is a robust approach to metabolic engineering.

This strategy has been successfully applied to *S. spinosa* for spinosad optimization. Glycosylation of the spinosad’s macrolactone core requires a four-enzyme cascade (Gtt, Gdh, Kre, and SpnG) responsible for L-rhamnose biosynthesis and attachment. Tao et al. (2018) demonstrated that chromosomal amplification of *gtt*, *gdh*, and *kre* genes elevated rhamnose pathway throughput, resulting in a 48% increase in spinosad yield. This breakthrough highlights the critical role of glycosylation precursor supply in spinosyn biosynthesis, providing a template for further refinement of the pathway.

Secondary metabolite precursors are crucial biochemical nodes that connect primary metabolism with specialized biosynthetic pathways. In polyketide antibiotic biosynthesis, exemplified by spinosad production, acetyl-CoA and propionyl-CoA are the essential starter and extender units, respectively. These precursors are generated via central carbon metabolism pathways, establishing a direct metabolic coupling between primary metabolic fluxes and secondary metabolite output (Wu et al., 2021). Recent studies have asserted that engineering microbial systems to augment intracellular pools of acetyl-CoA and propionyl-CoA substantially improves the titers of polyketide derivatives, including spinosyns (Wang et al., 2021; Liao et al., 2022).

Our research group systematically constructed a genome-scale metabolic network for spinosad biosynthesis via integrated multiomics analyses (genomics, proteomics, transcriptomics, and metabolomics) combined with clustered regularly interspaced short palindromic repeats (CRISPR)–Cas9-mediated genome editing (Xia et al., 2024; Liu et al., 2020; Rang et al., 2020). Comparative multiomics profiling revealed critical metabolic limitations in wild-type (WT) *S. spinosa*: key primary metabolic pathways supplying spinosad precursors exhibited suboptimal gene expression and metabolite flux, and the propionyl-CoA carboxylase (PCC)–catalyzed conversion of propionyl-CoA to methylmalonyl-CoA, a critical step for spinosad carbon chain elongation, was identified as a rate-limiting node. Both malonyl-CoA and methylmalonyl-CoA, essential extender units for PKS assembly, were chronically undersupplied in native metabolic states.

Spinosad, a highly valued polyketide insecticide produced by *Saccharopolyspora spinosa*, has garnered significant attention for its environmental safety and broad-spectrum activity. However, optimizing spinosad production remains a key challenge in industrial microbiology, with metabolic flux regulation and precursor availability emerging as critical bottlenecks in its biosynthetic pathway. Previous studies have highlighted the importance of primary metabolic pathways in supporting polyketide biosynthesis, yet the specific roles of key regulatory subunits–such as those of the propionyl-CoA carboxylase (PCC) complex–in modulating precursor flux for spinosad production remain underexplored.

Metabolic engineering strategies, including targeted genetic modifications and metabolic pathway remodeling, have shown promise in enhancing secondary metabolite production in Actinomycetes. Among these, CRISPR interference (CRISPRi)-mediated gene repression has emerged as a precise tool for manipulating metabolic networks, while integrative multi-omics analyses offer insights into global metabolic rewiring associated with production optimization. Additionally, strategic supplementation of essential nutrients has been demonstrated to alleviate precursor limitations, further supporting the potential of combinatorial approaches in yield improvement.

Against this backdrop, the present study focuses on elucidating the regulatory roles of PCC subunits (Kim et al., 2025), with particular emphasis on pccB1, in spinosad biosynthesis. By integrating CRISPRi-mediated metabolic modulation (Schultenkämper et al., 2020; Supplementary Table 1), proteomic profiling of pathway remodeling, and targeted nutrient supplementation, we aim to unravel the mechanisms by which precursor flux is governed and to develop a robust engineering strategy for enhancing spinosad productivity. Such efforts not only contribute to a deeper understanding of metabolic regulation in *S. spinosa* but also provide a framework for optimizing polyketide antibiotic production in Actinomycetes more broadly.

## 2 Materials and methods

### 2.1 Strains, primers, plasmids, medium, and growth conditions

The strains, plasmids, and primers used in this study are listed in Table 1. *S. spinosa* CCTCC M206084 was initially activated on corn starch medium (CSM) solid agar at 30 °C for 48 h. *Escherichia coli* strains were routinely cultured in Luria–Bertani medium, with antibiotic supplementation (when required) based on plasmid selection markers. Tryptic soy broth (TSB) and brain heart infusion (BHI) solid media were specifically used to comparatively analyze the sporulation capacities of WT and recombinant strains. Bacterial conjugation between *S. spinosa* and *E. coli* was performed using R6 medium, as previously described (Liu et al., 2021). For spinosad production analysis, seed cultures were inoculated into synthetic fermentation medium (SFM), with amino acid supplementation (1 mg/mL final concentration) where specified. All liquid cultures of the strains were incubated in 300 mL flasks containing 50 mL of medium, and all cultivation experiments were conducted in triplicate under aseptic conditions. The composition of the medium is listed in the Supplementary material (Supplementary: Composition of Culture Media). The relevant information pertaining to the plasmids has been deposited in Benchling.

**TABLE 1:** Strains and plasmids used in this study.

Strains	Relative description	Sources
*S. spinosa* (CCTCC M206084)	The original strain producing spinosad	Bought from CCTCC
*E. coli* DH5α	Host for general cloning	Bought from Thermo Fisher
*E. coli* S17	Donor strains for conjugation	Bought from Beyotime
*E. coli* BL21(DE3)	Host for heterologous expression	Bought from TaKaRa
*S. spinosa-pccB1*	*S. spinosa* harboring pOJ260**-***P*_*ermE*_**-***pccB1*	This work
*S. spinosa-*dCas9**-***pccB1*	*S. spinosa* harboring pSET**-**dCas9**-***pccB1*	This work
*E. coli* BL21(DE3)-pET-28a-*pccB1*	*E.coli* BL21(DE3) containing the plasmid pET-28a-*pccB1*	This work
**Plasmids**		
pOJ260	*E. coli*–cloning vector containing pUC18 replicon, oriT, and Apr-R	Bought from NovoPro
pSET-dCas9	Derived from pSET-152 harboring the dCas9 expression cassette, including the ermE*p promoter, the *dCas9* gene, and the *fd* terminator	Lab store
pET-28a	Expression plasmid for expressing the target protein AmpR	Lab store
pOJ260-*P*_*ermE*_-*pccB1*	*P*_*ermE*_-*pccB1* inserted into pOJ260 by *Hin*d III and *Eco*R I	This work
pSET-dCas9-*pccB1*	sgRNA of *pccB1* inserted into pSET-dCas9 by *Eco*R I and *Spe* I	This work
pET-28a-*pccB1*	*pccB1* inserted into pET-28a by *Eco*R I and *Hin*d III	This work

(pET28A,^1^

pOJ260,^2^

pSET-dCas9)^3^.

### 2.2 Construction of genetically modified strains

This study focused on PCC, encoded by the *pcc* gene cluster comprising *pccA* (A0A2N3XQX1), *pccB1* (A0A2N3XWG9), and *pccB2* (A0A0N3Y507). The strain engineering workflow comprised two parallel strategies: (1) overexpression strain construction and (2) CRISPRi strain development. A representative protocol for manipulating the *pccB1* gene is described below. (1) Overexpression strain construction: The *pccB1* gene (UniProt ID: A0A2N3XWG9) was polymerase chain reaction (PCR)–amplified from *S. spinosa* genomic DNA using primers *pccB1*-F/*pccB1*-R (Table 2). Simultaneously, the constitutive *P*_*ermE*_ promoter was amplified from the plasmid pOJ260-*P*_*ermE*_ using primers *P*_*ermE*_-F/*P*_*ermE*_-R. These fragments were fused via overlap extension PCR (Supplementary Figure 1) to generate the *P*_*ermE*_-*pccB1* transcriptional unit. The fusion product was subsequently digested with *Eco*R I/*Hind* III (Thermo Scientific) and ligated into a similarly restricted pOJ260 backbone (Supplementary Figure 2), yielding the recombinant plasmid pOJ260-*P*_*ermE*_-*pccB1*. 2. CRISPRi strain construction: For targeted gene suppression, a 200-bp sgRNA-*pccB1* cassette was amplified from the pSET-dCas9 template using primers *pccB1*-sgRNA-F/sgRNA-R (Table 2). This fragment was cloned into *Spe* I/*Eco*R I sites of pSET-dCas9 using DNA ligase, generating the CRISPRi plasmid pSET-dCas9-*pccB1* (Supplementary Figure 3). Both recombinant plasmids were introduced into WT *S. spinosa* via *E. coli*-mediated conjugation. Engineered strains were obtained via antibiotic selection (apramycin 50 μg/mL for pOJ260 derivatives; thiostrepton 25 μg/mL for pSET derivatives) (Cao et al., 2024).

**TABLE 2:** Nucleotide sequences of primers.

Primers	Sequences (5′→ 3′)
Apr-F	TTTGCAAGCAGCAGATTACG
Apr-R	ATCCGTCGACCTGCATACTA
*P*_*ermE*_-F	GCTAAGCTTCTGGACTTCTAGAGCTAGCCTCG (*Hin*d III)
*P*_*ermE*_-R	ACTGCGGTCATCGGATCCTCGCATGCCGGTCG ACTCTAGA
*pccB1*-F	TCTAGAGTCGACCGGCATGCGAGGATCCGATG ACCGCAGT
*pccB1*-R	GCTGAATTCTGCCCTGAAATTCCGGTTAC(*Eco*R I)
*pccB1*-sgRNA-F	TTGGACTAGTCGGAGAGCGACCAGGAACAGG TTTTAGAGCTAGAAATA(*Spe* I)
sgRNA-R	TAGAATTCGGGTGTACATCCAGTAATG(*Eco*R I)
*pccB1*-RT-F	CGTGCCTACGATGTCAAGCC
*pccB1*-RT-R	GCCGAAAGCGAGTCCAGA
*pccB2*-RT-F	CAGCGTGCCTACGATGTCA
*pccB2*-RT-R	CGAGACCGACCACGATGTT
*spnA*-RT-F	GACTGTCCTGCCGCCTAC
*spnA*-RT-R	GACATCAGCCAGGAAACCA
*spnB*-RT-F	AGCATCGCTCAAGGAGAACG
*spnB*-RT-R	CAGCCAGTTGCCACAGGTC
*spnC*-RT-F	AGGGCTGTGGAAACTGGTC
*spnC*-RT-R	CAGGAGATTTCCAGCAGCA
*spnD*-RT-F	CGGCTATCTGAAGAAGGTAA
*spnD*-RT-R	GAACTCCGAGATGGCGTC
*spnE*-RT-F	GAGGCACAGGCGATGGAC
*spnE*-RT-R	TAACCCGAAGGTGTAAGCCA
*spnF*-RT-F	CCGTTGCTGAACTCGGTCG
*spnF*-RT-R	GTCCGTTCGGCGACAAGGT
*spnH*-RT-F	GACAACACCGACTACAGGCA
*spnH*-RT-R	GGAAGGAATCCACTACCCAGA
*spnK*-RT-F	AACTTCCTCCGAACCACCAC
*spnK*-RT-R	TAGTGGGAGGCGAGCAAGTT
*spnQ*-RT-F	CCTGGCACTGAGTTCGCTTA
*spnQ*-RT-R	GTTGTAGGTGCCCAGTTCCA

### 2.3 Analysis of physiological and biochemical differences of strains

To evaluate the growth dynamics and morphological alterations, WT and engineered *S. spinosa* strains were precultured in CSM for 48 h at 30 °C, followed by submerged fermentation in SFM under identical conditions. Hyphal morphology was monitored daily using an electron microscope. For standardized protocols regarding biomass measurement and extracellular metabolite profiling, refer to supplementary: Analysis of Physiological and Biochemical Differences Among Strains (He et al., 2021).

### 2.4 Qualitative and quantitative detection of spinosad using high-performance liquid chromatography (HPLC) and liquid chromatography (LC)–mass spectrometry (MS)/MS

Fermentation broths (10-days old, 30 °C) from triplicate biological replicates were extracted with methanol (1:3 v/v), after lyophilizing the methanol, ethyl acetate was employed for the back-extraction of spinosad (1:1 v/v). After solvent evaporation, the residues were reconstituted in methanol for HPLC and LC–MS/MS analysis. The spinosad pure product (50 mg/L), supplied by Merck, was used as the stock solution, and a gradient dilution was performed using a methanol solution. An Agilent 1290 HPLC system equipped with a C18 reverse-phase chromatographic column (such as ZORBAX Eclipse Plus C18, 4.6 × 150 mm, 5 μm) was used, and the mobile phase comprised methanol:acetonitrile:ammonium acetate in a volumetric ratio of 42:42:16. The column temperature was 30 °C, the flow rate was 1.0 mL/min, the injection volume was 10 μL, and the detection wavelength was 246 nm. Linear regression was performed with the spinosad peak area against the concentration (mg/L) to obtain the standard curve equation. MS conditions were as follows: electrospray ionization source (ESI±), ion source temperature 500 °C, spray voltage ± 4500 V, curtain gas 35 psi, and collision gas (CAD) 8 psi. The target compounds were detected using the multiple reaction monitoring mode.

### 2.5 RNA extraction and quantitative reverse transcription (qRT)-PCR analysis

Total RNA was isolated from Day 2/4/6 SFM cultures using TRIzol^®^ (Invitrogen), followed by DNase I treatment (Takara). PrimeScript RT Master Mix (TaKaRa) was used for cDNA synthesis. qRT-PCR was performed on a QuantStudio 5 System (Applied Biosystems) with SYBR^®^ Green chemistry using *16S rRNA* (F: 5′-AGAGTTTGATCCTGGCTCAG-3′; R: 5′-GGTTACCTTGTTACGACTT-3′) as the endogenous control (Liu et al., 2020). Detailed procedures are described in the supplementary (Supplementary: RNA Isolation and Quantitative Real-Time PCR Analysis).

### 2.6 Determination of intracellular propionyl-coA and methylmalonyl-coA concentrations in recombinant strains

The WT and recombinant strains were fermented and cultured, and 5 mL of the SFM fermentation broth on the 2nd, 4th, and 6th days was used for testing. The sample supernatant was used to determine the concentrations of propionyl-CoA and methylmalonyl-CoA according to the instructions provided in the enzyme-linked immunosorbent assay kit. Sample processing and detection protocol: 5 mL of SFM fermentation broth collected at different time points (2 d, 4 d, and 6 d) was immediately placed on ice, washed three times with ultrapure water. The bacterial cells were ground in liquid nitrogen and then dissolved in phosphate buffered saline (PBS). 50 mM N-ethylmaleimide was added to inhibit degradation, and centrifuged at 12,000 × *g* for 15 min at 4 °C to obtain the supernatant. It was precipitated using prechilled 10% perchloric acid, incubated on ice for 10 min, and then centrifuged under the same conditions. The acidic extract was neutralized with 2 M KOH on an ice bath and centrifuged again to remove the precipitates. The resulting samples were aliquoted and stored at −80 °C for later use.

A standard curve was plotted using a series of concentrations (0.1–100 nM). Samples and standards were added to antibody-precoated plates and incubated at 37 °C for 1 h. After washing, the biotinylated secondary antibody horseradish peroxidase–streptavidin and the 3,3′,5,5′-tetramethylbenzidine substrate were sequentially added. The reaction was developed in the dark for 15 min and then terminated with 2 M H2SO4. The absorbance at 450 nm was measured, and the concentrations of propionyl-CoA and methylmalonyl-CoA were calculated using a four-parameter fitting curve.

### 2.7 Sodium dodecyl sulfate-polyacrylamide gel electrophoresis (SDS-PAGE) and western blot analysis

Strains were sonicated on ice in lysis buffer (50 mM Tris-HCl pH 8.0, 150 mM NaCl, 1% NP-40, 1 mM PMSF) and then centrifuged at 12,000 × *g* for 20 min at 4 °C to collect the supernatant. After determining the protein concentration using the bicinchoninic acid assay, 30 μg of total protein was mixed with 4 × Laemmli buffer and denatured at 95 °C for 5 min (Hassan et al., 2025). The samples were separated using 12% SDS-PAGE (constant voltage: 80 V for the stacking gel and 120 V for the resolving gel). Then, they were wet-transferred to a polyvinylidene fluoride membrane at 100 V and 4 °C for 90 min. The membrane was blocked with 5% skim milk in TBST (Tris- buffered saline + 0.1% Tween 20) at room temperature for 1 h, followed by sequential incubations: primary antibody: mouse anti-PccB1 polyclonal antibody (1:2000 in blocking buffer, overnight at 4 °C); secondary antibody: DyLight 488-conjugated goat antimouse IgG (1:5000 in TBST, 1 h at room temperature). After washing with TBST (three 10-min washes), signals were detected using a fluorescence imaging system. The fluorescence intensity of the target band (53 kDa) was quantified using the ImageQuant TL software (Tang et al., 2021).

### 2.8 Heterologous expression and western blot analysis of the PCCB1 protein

To verify the expression of *pccB1* in recombinant and wild-type strains, the heterologous expression vector pET28a-*pccB1* was constructed. Primers *pccB1*-F and *pccB1*-R were designed to amplify the *pccB1* gene from the genome of *S. spinosa*. After double digestion with *Eco*R I and *Hind* III, the *pccB1* gene was cloned into the plasmid pET28a to generate the recombinant vector pET28a-*pccB1*, which was then transformed into *E. coli* BL21. The recombinant strain was cultured in LB medium containing 50 μg/mL kanamycin to induce the expression of the PCCB1 protein. The expressed protein was injected into mice as an antigen, and Western blot analysis was subsequently performed to detect the expression of PCCB1 in both the recombinant and wild-type strains.

### 2.9 Proteomic sample processing

The 144-h SFM fermentation broth was used for proteomic testing. MS proteomics data were deposited in the ProteomeXchange Consortium via the PRIDE partner repository with the dataset identifier PXD062497. The detailed methods of proteomic treatment are listed in the Supplementary section (Supplementary: Proteomic Sample Processing, Proteomics data processing and statistical analysis).

### 2.10 Insecticidal toxicity test of spinosad

Spinosyns were extracted from the fermentation broth and incorporated into the artificial diet at a final concentration of 1 mg/L. The diet formulation contained (per liter) 60 g of flour, 120 g of soybean powder, 20 g of yeast, 13 g of agar, 2 g of ethyl p-hydroxybenzoate, and 1 g of sorbic acid, along with vitamin supplements (20 × vitamin C and 10 × vitamin B2 tablets). Control groups received equivalent volumes of sterile water instead of spinosyn extract. Both diets were dispensed into 24-well plates containing fourth-instar *Helicoverpa armigera* larvae (*n* = 24 per group). Mortality rates were recorded daily over a 4-days exposure period (Xia et al., 2024).

### 2.11 Statistical analysis

Error bars represent the standard error of the mean (based on *n* = 3 independent biological replicates). Student’s *t*-test was used to compare differences between groups (assuming normal distribution of data and homogeneity of variance). Statistical significance thresholds were set as *p* < 0.05 (*), *p* < 0.01 (**), and *p* < 0.001 (***).

## 3 Results

### 3.1 Detection of spinosad production in pCC-related engineered strains

Propionyl-CoA carboxylase catalyzes the ATP-dependent conversion of propionyl-CoA to methylmalonyl-CoA, a critical extender unit that synergizes with malonyl-CoA to assemble the polyketide backbone of spinosad. Because of the inherently low intracellular concentrations of these precursors in *S. spinosa*, a dual genetic strategy was employed to enhance PCC activity: (1) CRISPRi for targeted gene repression and (2) pOJ260 plasmid-mediated overexpression. Six engineered strains with modified *pccA*, *pccB1*, or *pccB2* expression were constructed and cultured for 14 days under standardized fermentation conditions.

**FIGURE 1:**
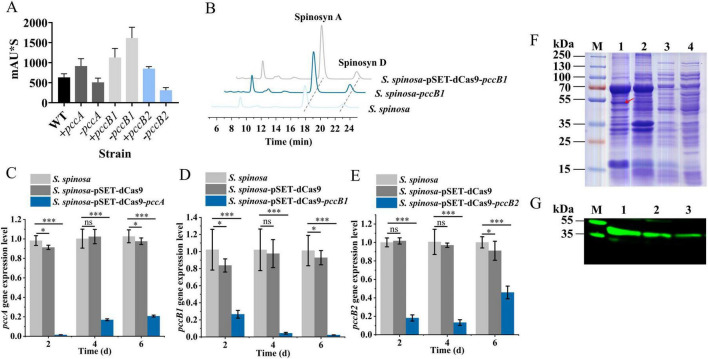
Screening and analysis of high-spinosad–producing strains. **(A)** Determination of spinosad production in WT and six engineered strains. **(B)** HPLC detection of spinosad in WT and *pccB1* engineered strain. **(C–E)** Transcription level determination of *pccA*, *pccB1*, and *pccB2* genes in *S. spinosa*, *S. spinosa*-pSET-dCas9 and inhibitory strains. **(F)** SDS-PAGE analysis of the heterologous expression of the PCCB1 protein. **(G)** Western blot detection of PCCB1 protein expression in WT and *pccB1* engineered strain. Statistical significance thresholds were set as *p* < 0.05 (*), *p* < 0.01 (**), and *p* < 0.001 (***).

Quantitative HPLC analysis revealed significant variations in spinosad production across strains (Figures 1A, B, Supplementary Figure 4). The CRISPRi-mediated repression of *pccB1* (strain *S. spinosa*-pSET-dCas9-*pccB1*) paradoxically led to a 2.6-fold increase in spinosad yield compared with the WT. In contrast, the overexpression of *pccA* and *pccB2* resulted in a 1.8- and 1.5-fold increase in production, respectively. The structure of spinosad was determined using high-resolution LC–MS (Supplementary Figure 5), with characteristic molecular ions that matched the reference spectra.

The observed production patterns were aligned with PCC’s proposed role in precursor supply: overexpression strains exhibited elevated methylmalonyl-CoA levels, consistent with enhanced PKS initiation and elongation efficiency. However, the unexpected hyperproduction in the *pccB1*-repressed strain suggests complex regulatory dynamics; follow-up analyses identified a compensatory regulation mechanism.

This counterintuitive finding highlights that *pccB1* is a potential central point of metabolic flux at which partial repression triggers cellular adaptation mechanisms that ultimately optimize spinosad biosynthesis. Further investigation of post-translational regulation and pathway crosstalk is critical for understanding this phenomenon.

### 3.2 Analysis of the reasons for the high spinosad production in *S. spinosa*-pSET-dCas9-*pccB1*

Quantitative RT-qPCR analysis confirmed the effective transcriptional repression of *pccB1* in CRISPRi strains (Figures 1C–E, Supplementary Figure 6), showing a reduction compared with the WT. To validate protein-level effects, a heterologous *pccB1* expression system was constructed (Supplementary Figure 7) and immunoblotting was performed with anti-PccB1 polyclonal antibodies generated from mouse cardiac serum. SDS-PAGE of recombinant protein extracts revealed distinct bands at the predicted 53 kDa molecular weight (Figure 1F). Subsequent western blot using DyLight 488–conjugated goat antimouse IgG secondary antibody demonstrated significantly higher fluorescence intensity in overexpression strains compared with the WT. In contrast, CRISPRi strains exhibited signal reduction (Figure 1G), confirming successful translational regulation.

**FIGURE 2:**
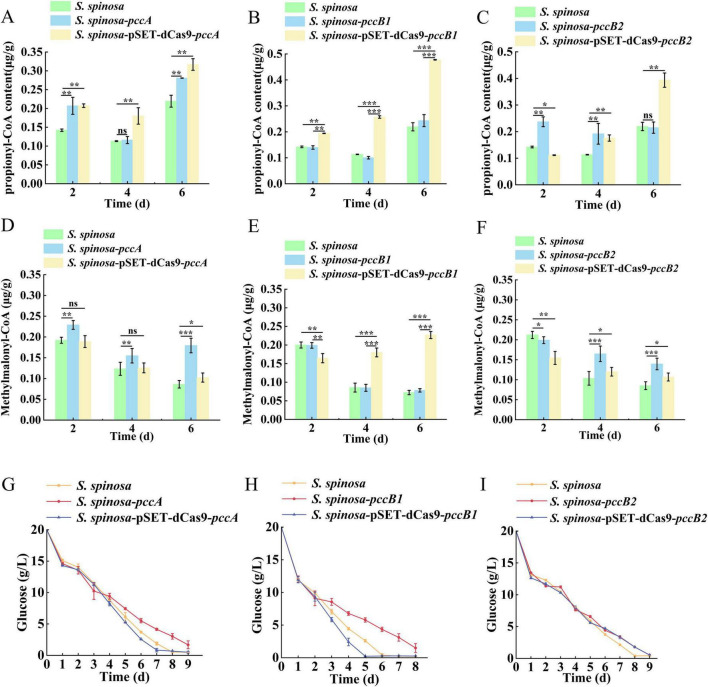
In-depth analysis of *S. spinosa*-pSET-dcas9-*pccB1* to determine the reasons for the high yield of the strain. **(A–F)** Determination of the content of propionyl-CoA and methylmalonyl-CoA in WT and engineered strains, the *Y*-axis represents the concentrations of propionyl-CoA and methylmalonyl-CoA in the strains, expressed as the weight of propionyl-CoA or methylmalonyl-CoA (μg) per weight of cell wet biomass (g). **(G–I)** Glucose consumption analysis of WT and engineered strains. Statistical significance thresholds were set as *p* < 0.05 (*), *p* < 0.01 (**), and *p* < 0.001 (***).

**FIGURE 3:**
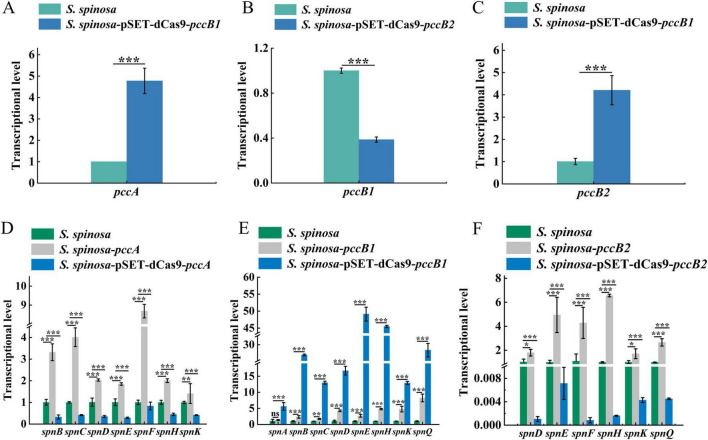
Transcriptional analysis of spinosad synthesis key genes in WT and engineered strains. **(A–C)** Transcription level determination of *pccA, pccB1* and *pccB2* genes in WT and inhibitory strains, respectively. **(D–F)** Transcription level determination of spinosad synthesis *spn* series genes in WT and engineered strains. Statistical significance thresholds were set as *p* < 0.05 (*), *p* < 0.01 (**), and *p* < 0.001 (***).

Dynamic changes in spinosad precursors were observed across engineered strains (Figures 2A–F). The *pccB1*-repressed strain accumulated higher concentrations of propionyl-CoA (Day 6) and methylmalonyl-CoA (Day 4) compared with the WT, despite reduced *pccB1* expression. Concurrently, glucose consumption rates increased in repression strains (Figures 2G–I), indicating enhanced carbon flux via these pathways. These paradoxical findings prompted the investigation of PCC subunit cross-regulation. Transcript analysis demonstrated reciprocal compensation: *pccA* and *pccB2* expression increased in *pccB1-*repressed strains, while *pccB1* repression in *pccB2*-repressed strains reduced *pccB1* transcripts (Figures 3A–C). This regulatory crosstalk likely maintains functional PCC holoenzyme assembly, as evidenced by the sustained production of methylmalonyl-CoA.

The transcriptional activation of PKS genes (*spnA–J*) in *pccB1*-repressed strains exceeded WT levels (Figures 3D–F). This coordinated upregulation of both precursor supply (methylmalonyl-CoA) and biosynthetic machinery (*spn* cluster) creates a feed-forward loop that drives spinosad hyperproduction. The metabolic rewiring appears to involve two compensatory mechanisms: subunit redundancy in PCC complex assembly, which allows *pccB2* to compensate for *pccB1* deficiency, and carbon flux redistribution via activation of the tricarboxylic acid (TCA) cycle.

These findings redefine the regulatory role of PCC in secondary metabolism, demonstrating that targeted repression of specific subunits can trigger global metabolic adaptations that paradoxically enhance antibiotic biosynthesis.

### 3.3 Proteomics reveals the reason for increased spinosad biosynthesis in *S. spinosa*-pSET-dCas9-*pccB1*

Comparative proteomic analysis of *S. spinosa*-pSET-dCas9-*pccB1* vs. the WT via the Kyoto Encyclopedia of Genes and Genomes pathway enrichment and eggNOG functional annotation revealed extensive metabolic remodeling (Supplementary Figure 8). The engineered strain exhibited a significant upregulation (>1.5-fold change, *p < 0.05*) of proteins linked to spinosad biosynthesis and energy metabolism, indicating a redirection of metabolic resources toward secondary metabolite production.

**FIGURE 4:**
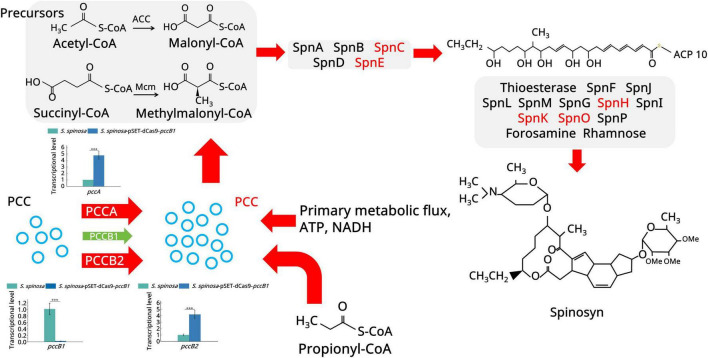
Spinosad secondary metabolism biosynthesis pathway. The figure illustrates the spinosad secondary metabolism biosynthesis pathway and the expression of the proteins involved. Red color indicates upregulated proteins. Green color indicates downregulated proteins. Different panels represent different stages in the biosynthesis process of spinosad. Statistical significance thresholds were set as *p* < 0.05 (*), *p* < 0.01 (**), and *p* < 0.001 (***).

Focusing on spinosad precursor pathways (Figure 4), an 8.7-fold increase was observed in PCC α-subunit (PccA) expression, driving the accelerated conversion of propionyl-CoA to methylmalonyl-CoA. This catalytic surge facilitated dual metabolic routing: channeling of methylmalonyl-CoA into the TCA cycle via succinyl-CoA (1.8-fold flux increase) and its enhanced incorporation into polyketide backbones via the coordinated activation of macrolide synthases (12-, 14-, and 16-acetyl transferases upregulated 3.1–4.5 fold) (Supplementary Figure 9).

**FIGURE 5:**
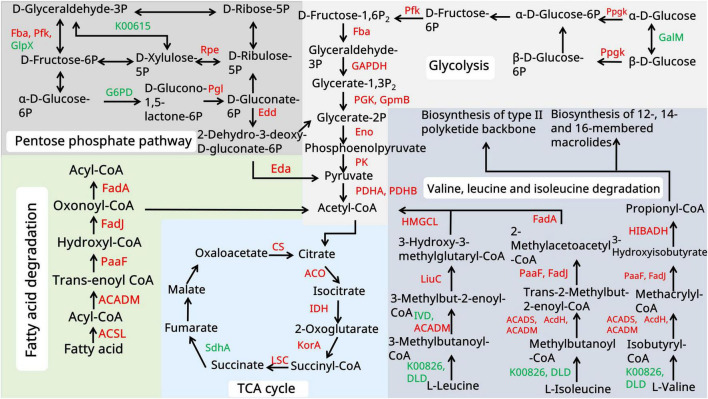
Primary metabolism analysis of the WT and *pccB1* inhibitory strains. The figure depicts the key spinosad biosynthesis-related primary metabolism pathways and protein expression associated with them. Red color indicates upregulated proteins. The green color suggests downregulated proteins. Different color panels represent different metabolic pathways.

Global carbon metabolism analysis (Figure 5) demonstrated a multilevel phenomenon. In acetyl-CoA amplification, pyruvate kinase (2.97-fold upregulation) and L-serine dehydratase (5.2-fold upregulation) enhanced pyruvate synthesis. In oxidative decarboxylation, the upregulation of the pyruvate dehydrogenase complex (7.4-fold) increased the acetyl-CoA yield. In TCA cycle activation, key enzymes, including aconitate hydratase (2.3-fold upregulation), isocitrate dehydrogenase (2.6-fold upregulation), and 2-oxoglutarate ferredoxin oxidoreductase (3.9-fold upregulation), collectively increased ATP generation (1.7-fold) and the production of metabolic intermediates. The fatty acid degradation pathway showed remarkable activation: long-chain acyl-CoA synthetase (5.2-fold upregulation), acyl-CoA oxidase (5.3-fold upregulation), and enoyl-CoA hydratase (3.9-fold upregulation). This enzymatic triad enhanced β-oxidation efficiency, converting stored lipids into acetyl-CoA at higher rates than in the WT.

The coordinated upregulation of complementary acetyl-CoA production routes, spanning glycolysis, amino acid catabolism, and lipid degradation, establishes the metabolic flexibility of the strain. This system-level adaptation not only sustains energy demands via enhanced spinosad biosynthesis but also maintains redox balance by reinforcing the TCA cycle. The *pccB1* repression appears to trigger a global metabolic state that favors secondary metabolism, as revealed by proteomic data, which show that many of the upregulated proteins are directly associated with precursor supply or energy transduction.

### 3.4 Phenotypic analysis of *S. spinosa*-pSET-dCas9-*pccB1*

**FIGURE 6:**
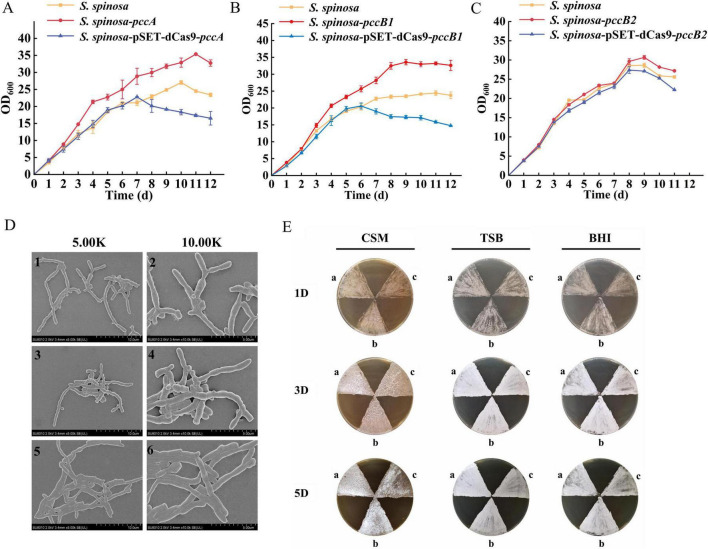
Phenotypic observation and biomass detection of strains. **(A–C)** Biomass analysis of the WT and engineered strains. **(D)** Electron microscopic observation of the morphology of WT and *pccB1* engineered strains. 1–2: WT; 3–4: *pccB1* overexpression strain; 5–6: *pccB1* inhibitory strain. **(E)** Spore generation of the WT and *pccB*1 engineered strains. Comparison of the spore-producing ability of *pccB1* engineered strains on CSM, TSB, and BHI solid media. a. WT strain; b. *pccB1* overexpression strain; c. *pccB1* inhibitory strain.

Analysis of growth kinetics demonstrated distinct physiological adaptations in *S. spinosa*-pSET-dCas9-*pccB1* compared with the WT. The engineered strain exhibited a shorter stationary phase and an earlier entry into the decline phase (Figures 6A–C), which correlated with accelerated glucose consumption rates. This metabolic prioritization was accompanied by a reduction in the final biomass, indicating a reallocation of resources toward secondary metabolism at the expense of vegetative growth.

Scanning electron microscopic examination of 48-h mycelia revealed that the hyphal morphology and branching patterns were conserved between strains (Figure 6D). Nonetheless, sporulation assays on CSM, TSB, and BHI media showed significantly reduced spore formation in the CRISPRi strain (Figure 6E). In addition, proteomic profiling identified complete suppression of the spore germination protein YaaH, coupled with the downregulation of the developmental regulators WhiG and SsgA. This phenotypic shift suggests the adoption of a metabolically oriented survival strategy, favoring the rapid utilization of precursors over developmental competency.

The observed growth–sporulation trade-off aligns with the overflow metabolism hypothesis, which posits that enhanced carbon flux through acetyl-CoA nodes redirects cellular resources from differentiation programs to secondary metabolite synthesis. The concurrent loss of sporulation efficiency and activation of spinosad biosynthetic genes (*spn* cluster) signifies hierarchical metabolic prioritization under genetic perturbation.

### 3.5 Multiamino acid supplementation further optimizes spinosad production

**FIGURE 7:**
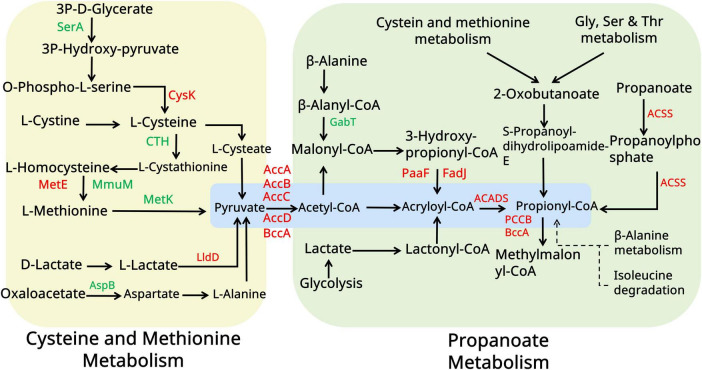
Amino acid metabolism analysis of the WT and *pccB1* inhibitory strains. The figure illustrates the key spinosad biosynthesis-related amino acid metabolism pathways and protein expression associated with them. Red color indicates upregulated proteins. The green color indicated downregulated proteins. Yellow and green panels represent different metabolic pathways. Blue panels represent the core metabolic substance connected to spinosad production.

**FIGURE 8:**
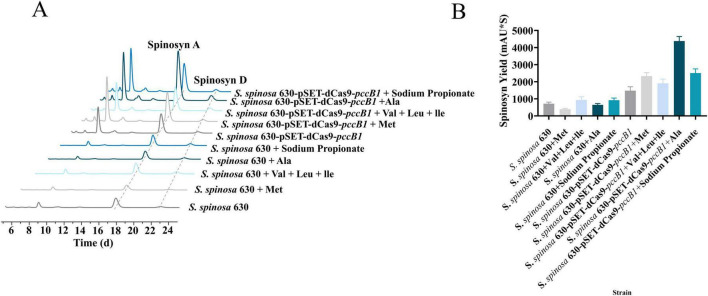
Amino acid optimization for spinosad production. **(A)** Spinosad peak detection of WT and engineered strains using HPLC with different amino acids. **(B)** Spinosad yield determination of WT and engineered strains using HPLC with different amino acids.

Building upon proteomic findings that revealed a significant upregulation of amino acid metabolic enzymes (log2FC > 1.5, *p < 0.01*) in the *pccB1*-repressed strain, a metabolic engineering strategy was devised to enhance precursor availability for spinosad biosynthesis. Pathway analysis identified three key metabolic nodes capable of generating acetyl-CoA and propionyl-CoA precursors: cysteine/methionine metabolism, branched-chain amino acid (valine/leucine/isoleucine) degradation, and propanoate metabolism (Figure 7). Targeted supplementation of 1 mg/mL valine, leucine, isoleucine, methionine, alanine, or propionate during mid-log phase fermentation resulted in differential enhancement of spinosad production (Figure 8A). The *pccB1*-repressed strain exhibited superior precursor utilization efficiency, with alanine supplementation yielding maximal titers of 590 ± 31 mg/L, a 6.2-fold increase over WT baseline production (95 ± 13 mg/L; Figure 8B, Supplementary Figure 10).

The exceptional performance of alanine stems from its dual metabolic fates: direct conversion to pyruvate via alanine dehydrogenase, subsequent oxidative decarboxylation to acetyl-CoA via the pyruvate dehydrogenase complex, and anaplerotic entry into propionyl-CoA synthesis via methylmalonyl-CoA mutase. This metabolic flexibility synergizes with the enhanced amino acid catabolic capacity of the strain, as evidenced by the upregulation of branched-chain amino acid transaminases and methionine γ-lyases. Upon supplementation of alanine to the *pccB1*-repressed strain, spinosad yield was significantly enhanced. This improvement is likely attributed to the redirection of exogenous alanine toward optimizing metabolic flux operation, thereby refining precursor distribution within the strain.

**FIGURE 9:**
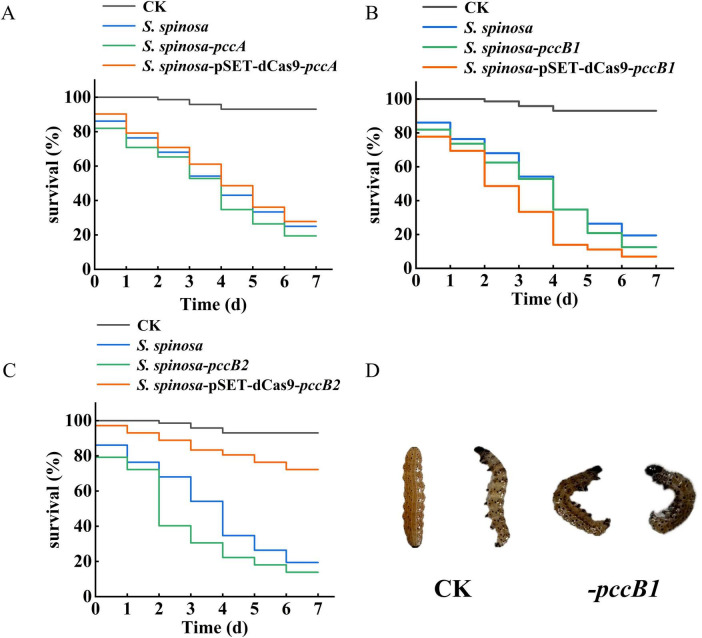
Insecticidal toxicity test of spinosad. **(A–C)** Survival rate of *Helicoverpa armigera* during insecticidal toxicity test of spinosad. **(D)** Toxicological characteristics observation of *H. armigera*.

To confirm the functional enhancement of spinosad production, standardized bioassays were performed using third-instar *H. armigera* larvae (Figures 9A–C). The *pccB1*-repressed strain exhibited significantly increased insecticidal efficacy (Figure 9D). Symptomatology analysis revealed rapid-onset neurotoxic effects consistent with spinosad’s mode of action, including neuromuscular hyperexcitation, progressive paralysis, and synaptic dysfunction. These observations agree with spinosad’s known disruption of insect neuronal signaling, confirming that the enhanced titers in the *pccB1*-repressed strain are directly correlated with functional bioactivity. The dose-dependent mortality curves and symptom progression validated the quantitative and qualitative improvements in spinosad production.

## 4 Discussion

Spinosad demonstrates broad-spectrum insecticidal activity against agriculturally significant pests across six orders, including Lepidoptera, Thysanoptera, Coleoptera, Hymenoptera, and Diptera. Spinosad’s unique dual-mode neurotoxic action–simultaneously targeting nicotinic acetylcholine receptors and γ-aminobutyric acid–gated ion channels–ensures exceptional target specificity with minimal toxicity to nontarget organisms. In Actinomycete secondary metabolism, PKSs construct molecular scaffolds via iterative decarboxylative condensations. PCC serves as the metabolic gate for methylmalonyl-CoA production, a critical C3 extender unit that comprises the macrolide core of spinosad (Wang et al., 2021). The PCC holoenzyme operates via the coordinated specialization of its subunits. The α-subunit (PccA) serves as a biotin-dependent ATPase that mediates bicarbonate fixation. The biotin carboxyl carrier (BCC) domain activates the CO between catalytic sites. The β-subunit (PccB) functions as a transcarboxylase, transferring carboxyl groups to propionyl-CoA. This tripartite architecture enables sequential catalysis: PccA activates CO via biotin carboxylation, BCC translocates carboxyl-biotin to the β-subunit, and PccB transfers CO to propionyl-CoA and methylmalonyl-CoA. The resulting methylmalonyl-CoA pools integrate with acetyl-CoA/malonyl-CoA via PKS loading domains, initiating polyketide chain elongation through decarboxylative Claisen condensations. This biochemical logic positions PCC as a critical flux control point; each catalytic cycle generates one NADPH and directs the carbon flux toward either TCA cycle anaplerosis or secondary metabolite biosynthesis.

Acetyl-CoA plays dual roles in spinosad biosynthesis: it contributes to the polyketide backbone via iterative condensation with propionyl-CoA, methylmalonyl-CoA, and malonyl-CoA and also functions as a central metabolic nexus that links glycolysis, β-oxidation, and amino acid catabolism. This biochemical relationship underscores the importance of enhancing acetyl-CoA metabolic pathways through targeted genetic interventions. Rewiring recent advances in metabolic engineering demonstrate that modulating transcriptional regulators of precursor nodes can overcome pathway bottlenecks (Pudhuvai et al., 2024). In *Streptomyces* CDK506, methylmalonyl-CoA scarcity limits FK506 titers by a constraint alleviated via *accR* deletion. Parallel studies in *S. avermitilis* have revealed that AccR, a TetR-family repressor, gates avermectin biosynthesis by binding *acc* operon promoters to block malonyl-CoA synthesis, allosterically inhibiting methylmalonyl-CoA mutase. These findings provide a blueprint for spinosad optimization using CRISPRa-mediated activation of acetyl-CoA carboxylase (*accA1* and *accB*), deletion of competing pathway regulators (Lyu et al., 2020).

Herein, six *pcc*-engineered *S. spinosa* strains (*pccA*, *pccB1*, and *pccB2* CRISPRi/overexpression variants) were constructed to investigate precursor-driven optimization of spinosad biosynthesis. Contrary to initial hypotheses, CRISPRi-mediated repression of *pccB1* (strain *S. spinosa*-pSET-dCas9-*pccB1*) showed a 2.6-fold spinosad yield enhancement–the highest among all engineered variants. Integrated multiomics analyses revealed that this paradoxical hyperproduction could be attributed to three compensatory mechanisms. *pccB1* repression triggered the upregulation of *pccB2* transcripts, suggesting functional redundancy within the PCC β-subunit family. Proteomic quantification demonstrated that PccA (α-subunit) expression increased, enhancing carboxyltransferase activity. Global pathway analysis revealed that upregulated proteins were associated with glycolysis and β-oxidation, driving acetyl-CoA synthesis. These findings challenge conventional metabolic engineering paradigms by demonstrating that partial enzyme complex disruption (*pccB1* repression) can enhance overall catalytic efficiency via subunit rebalancing. Metabolic flux is governed not solely by transcriptional regulators but by holoenzyme stoichiometry and isozyme compensation. Targeted perturbation of “noncritical” subunits may induce beneficial network-wide adaptations. The *pccB1* repression phenotype exemplifies metabolic elasticity, with the engineered systems self-optimizing flux distribution via inherent regulatory plasticity. This principle opens new avenues for minimal-intervention engineering strategies in PKS.

Metabolic network activation and precursor channeling proteomic profiling of the *pccB1*-repressed strain revealed coordinated upregulation of enzymes in three critical precursor-generating pathways: glycolysis, fatty acid β-oxidation, and amino acid catabolism. This multipathway activation established a metabolic boosting effect, increasing the flux rates of intracellular acetyl-CoA and propionyl-CoA. Capitalizing on this enhanced enzymatic capacity, a targeted amino acid supplementation strategy was implemented. Branched-chain and sulfur-containing amino acids (valine, leucine, isoleucine, and methionine) showed moderate improvements in spinosad yield. However, alanine supplementation resulted in maximal enhancement, achieving 590 ± 31 mg/L spinosad production in the *pccB1*-repressed strain, a 6.2-fold increase over the WT. This superiority can be explained by alanine’s streamlined metabolic conversion: direct deamination, alanine → pyruvate via alanine dehydrogenase.

The strain’s rewired metabolism demonstrates three synergistic effects. Amplified precursor pools: Acetyl-CoA and methylmalonyl-CoA concentrations exceeded the WT levels. Energy–metabolite coupling: The activation of the TCA cycle generated more ATP to fuel PKS activity. Flux partitioning optimization: The supplemented alanine was channeled into spinosad biosynthesis in the WT, demonstrating enhanced precursor utilization efficiency. This engineered metabolic network creates a self-reinforcing cycle, with the elevated precursor flux stimulating PKS activity and the consumption of PKS maintaining favorable thermodynamics for continued precursor synthesis.

## Data Availability

The proteomics data presented in the study are publicly available. This data can be found here: https://www.proteomexchange.org/, dataset identifier PXD062497.

## Footnotes

1
https://benchling.com/s/seq-Q1wXccSQLfcBnlo8K0iP?m=slm-nrg50ICkUoPOVos6H15d

2
https://benchling.com/s/seq-zzYUXZaOy9jeM0FSH71r?m=slm-eBcQvkR4xcoGHkhreHfb

3
https://benchling.com/s/seq-XduK7CW9ATHTJ9PHy5vT?m=slm-xC7EwyNFrMQ8AGkykijw
