# Supplementary material for: Coordinated regulation of propionyl-CoA carboxylase subunits drives precursor flux optimization in spinosad production
Source: Front Microbiol. 2025 Sep 16;16:1643527. doi: 10.3389/fmicb.2025.1643527 (PMC12481512; doi:10.3389/fmicb.2025.1643527)
Supplement: Supplementary file 1 [file Supplementary_file_1.docx]

**Coordinated Regulation of Propionyl-CoA Carboxylase Subunits Drives Precursor Flux Optimization in Spinosad Production**

Ziyuan Xia^a, #^, Xiaomin Li^a, #^, Xirong Liu^b^, Li Cao^a^, Duo Jin^a^, Zirui Dai^a^, Baolong Bai^a^, Qian Liu^a^, Jie Rang^a^, Zirong Zhu^a,^ *, Liqiu Xia^a,^ *

^a^ Hunan Provincial Key Laboratory for Microbial Molecular Biology, State Key Laboratory of Developmental Biology of Freshwater Fish, College of Life Science, Hunan Normal University, Changsha, Hunan 410081, China.

^b^ Hunan Norchem Pharmaceutical Co., Ltd. Changsha, Hunan 410205, China.

Ziyuan Xia ^#^ and Xiaomin Li ^#^ contributed to this work equally.

*Corresponding author: Zirong Zhu, E-mail: 1832499112@qq.com

*Corresponding author: Liqiu Xia, E-mail: [xialq@hunnu.edu.cn](mailto:xialq@hunnu.edu.cn)

**Table S1. Engineered strains in this study**

| **Number** | **Gene** | **Strain** | **Method** |
| --- | --- | --- | --- |
| 1 | *pccA* | *S. spinosa*-*pccA* | Overexpression |
| 2 | *pccA* | *S. spinosa*-pSET-dCas9-*pccA* | Repression |
| 3 | *pccB1* | *S. spinosa*-*pccB1* | Overexpression |
| 4 | *pccB1* | *S. spinosa*-pSET-dCas9-*pccB1* | Repression |
| 5 | *pccB2* | *S. spinosa*-*pccB2* | Overexpression |
| 6 | *pccB2* | *S. spinosa*-pSET-dCas9-*pccB2* | Repression |

**Composition of Culture Media**

CSM solid medium (per liter): TSB 45 g, glucose 10 g, yeast extract 9 g, MgSO₄·7H₂O 2.2 g.

LB medium (per liter): yeast extract 5 g, tryptone 10 g, NaCl 10 g.

TSB solid medium (per liter): tryptic soy broth 30 g.

BHI medium (per liter): brain heart infusion 37 g.

R6 medium (per liter): sucrose 200 g, BHI 26 g, dextrin 10 g, casamino acids 1 g, K₂SO₄ 0.1 g, FeSO₄·7H₂O 0.1 g, MgSO₄·7H₂O 0.05 g, MnCl₂·4H₂O 0.001 g, ZnSO₄·7H₂O 0.001 g, sterile 1 mol/L MOPS 1 mL, 5 mol/L CaCl₂ 1 mL, 3.25 mol/L L-sodium glutamate 2 mL.

SFM medium (per liter): glucose 20 g, tryptone 4 g, yeast extract 4 g, KNO₃ 1 g, MgSO₄·7H₂O 0.5 g, K₂HPO₄·3H₂O 0.5 g, FeSO₄ 0.01 g; adjusted to 1 L with ultrapure water, pH 7.2.

**Analysis of Physiological and Biochemical Differences Among Strains​**

Physiological and biochemical differences between strains were analyzed using SFM fermentation broths samples. The specific detection indicators and experimental procedures were as follows:​

1. Growth curve determination​

The optical density (OD₆₀₀) of the strains was measured using a spectrophotometer every 24 hours throughout the fermentation period. Three parallel samples were set for each strain, and the average OD₆₀₀ value was calculated to plot the growth curves, reflecting the dynamic growth trends of different strains.​

2. Mycelium morphology observation​

For mycelium observation, 1 mL of 48-hour fermentation broth from each strain was collected and centrifuged at 6,000 × g for 5 minutes. The supernatant was discarded, and the mycelium pellet was washed with sterile distilled water 10 times to remove residual medium components. The cleaned mycelia were fixed in 2.5% glutaraldehyde solution (prepared with 0.1 M phosphate buffer, pH 7.2) at 4°C for 24 hours. After gradient dehydration with ethanol (30%, 50%, 70%, 90%, and 100%), the samples were dried using a critical point dryer, sputter-coated with gold, and observed under a scanning electron microscope (SEM) to compare the morphological characteristics of mycelia between the wild-type strain and recombinant strains.​

3. Glucose concentration detection in fermentation broth​

The glucose concentration in the fermentation broth was determined using a commercial glucose detection kit. The assay was performed according to the kit instructions, with three technical replicates for each sample. The absorbance was measured at 505 nm wavelength, and glucose concentration was calculated using a standard curve generated with known glucose standards.​

4. Spore formation observation​

To evaluate spore formation ability, the strains were streaked onto CSM, BHI, and TSB solid media respectively, and incubated at 30°C for 5 days. The morphology of spores were observed, and the spore-forming capacity was compared across different media types.

**RNA Isolation and Quantitative Real-Time PCR Analysis​**

Total RNA was extracted from 1 mL of SFM fermentation cultures harvested on days 2, 4, and 6 of cultivation. RNA isolation was performed using TRIzol® Reagent following the manufacturer’s standard protocol: briefly, 1 mL of TRIzol® was added to each culture sample, vortexed vigorously for 30 seconds, and incubated at room temperature for 5 minutes to allow complete lysis of cells. Chloroform (200 μL) was then added, the mixture was shaken vigorously for 15 seconds, and incubated at room temperature for 3 minutes. After centrifugation at 12,000 × g for 15 minutes at 4°C, the upper aqueous phase (containing RNA) was carefully transferred to a new RNase-free tube. An equal volume of isopropanol was added to precipitate RNA, followed by incubation at -20°C for 30 minutes and centrifugation at 12,000 × g for 10 minutes at 4°C. The RNA pellet was washed twice with 75% ethanol (prepared with RNase-free water), air-dried for 5 minutes, and resuspended in 50 μL of RNase-free water.​

To eliminate genomic DNA contamination, the isolated RNA was treated with DNase I. The reaction system (20 μL) contained 2 μg of total RNA, 2 μL of 10× DNase I Buffer, 1 μL of DNase I (10 U/μL), and RNase-free water to volume. The mixture was incubated at 37°C for 30 minutes, followed by the addition of 1 μL of 0.5 M EDTA to terminate the reaction at 65°C for 10 minutes. RNA concentration and purity were determined using a NanoDrop™ 2000 Spectrophotometer (Thermo Fisher Scientific), with acceptable A260/A280 ratios ranging from 1.8 to 2.0. RNA integrity was verified by 1% agarose gel electrophoresis, showing rRNA bands.​

First-strand cDNA synthesis was performed using PrimeScript™ RT Master Mix in a 20 μL reaction system: 1 μg of DNase I-treated RNA, 4 μL of 5× PrimeScript™ RT Master Mix, and RNase-free water to volume. The reverse transcription program was set as follows: 37°C for 15 minutes (cDNA synthesis), 85°C for 5 seconds (enzyme inactivation), and holding at 4°C. The synthesized cDNA was diluted 10-fold with RNase-free water and stored at -20°C until use.​

Quantitative real-time PCR (qRT-PCR) was carried out on a QuantStudio 5 Real-Time PCR System (Applied Biosystems) using SYBR® Green chemistry (TB Green® Premix Ex Taq™ II, Takara). Each 20 μL reaction mixture contained 10 μL of 2× TB Green® Premix Ex Taq™ II, 0.4 μL of forward primer (10 μM), 0.4 μL of reverse primer (10 μM), 2 μL of diluted cDNA template, and 7.2 μL of RNase-free water. The 16S rRNA gene was used as the endogenous reference gene, with primer sequences: forward (F): 5′-AGAGTTTGATCCTGGCTCAG-3′ and reverse (R): 5′-GGTTACCTTGTTACGACTT-3′ (synthesized by Sangon Biotech). The qRT-PCR cycling conditions were: initial denaturation at 95°C for 30 seconds, followed by 40 cycles of 95°C for 5 seconds (denaturation) and 60°C for 30 seconds (annealing and extension). A melting curve analysis was performed after amplification (95°C for 15 seconds, 60°C for 1 minute, and gradual heating to 95°C) to confirm the specificity of PCR products.​

All reactions were run in triplicate technical replicates for each sample, and three independent biological replicates were included for each time point (days 2, 4, and 6).

**Proteomic Sample Processing**

(1) Transfer the protein solution sample to a 10 kDa ultrafiltration tube and centrifuge at 12,000 × g for 20 minutes.
(2) Add 200 μL of Buffer 1 (8 M urea/100 mM Tris-HCl, pH 8.5) to each ultrafiltration tube to fully dissolve and denature the protein.
(3) Add 20 μL of 100 mM dithiothreitol (DTT) solution and incubate at 37 °C for 2 hours to reduce disulfide bonds.
(4) Add 20 μL of 500 mM iodoacetamide (IAA) solution and react at room temperature in the dark for 15 minutes.
(5) Centrifuge the reduced and alkylated protein solution at 12,000 × g for 20 minutes, then discard the solution in the bottom of the collection tube. Add 200 μL of Buffer 1 and repeat centrifugation twice.
(6) Add 200 μL of Buffer 2 (8 M urea/100 mM Tris-HCl, pH 8.0), centrifuge at 12,000 × g for 20 minutes, discard the solution in the bottom of the collection tube, and repeat this step once.
(7) Add 200 μL of 25 mM ammonium bicarbonate solution, centrifuge at 12,000 × g for 20 minutes, discard the solution in the bottom of the collection tube, and repeat this step twice.
(8) Replace the collection tube with a new one, add 100 μL of trypsin solution (0.01 μg/μL), and incubate at 37 °C for 14 hours.
(9) Remove the ultrafiltration tube, centrifuge at 12,000 × g for 20 minutes, and collect the enzymatically hydrolyzed peptides.
(10) Add 100 μL of 25 mM ammonium bicarbonate solution to the ultrafiltration tube, centrifuge at 12,000 × g for 10 minutes, collect the solution from the bottom of the tube, and combine it with the previously collected solution.

**Proteomics data processing and statistical analysis**

1. Data Preprocessing: Raw mass spectrometry files were processed using Proteome Discoverer with default parameters. Peptide identification was performed against the UniProt Reference Proteome database. Contaminants and reverse decoy sequences were removed.

2. Normalization: We utilized Median Centering across all samples to ensure comparability. Normalization was validated via density plots and PCA before downstream analysis.

3. Statistical Analysis: Differential expression analysis was conducted using Limma, Perseus.Proteins with a fold change > 1.5 and p-value < 0.05 (adjusted by Benjamini-Hochberg) were deemed significant.Functional enrichment analysis was performed via Metascape using GO and KEGG databases.


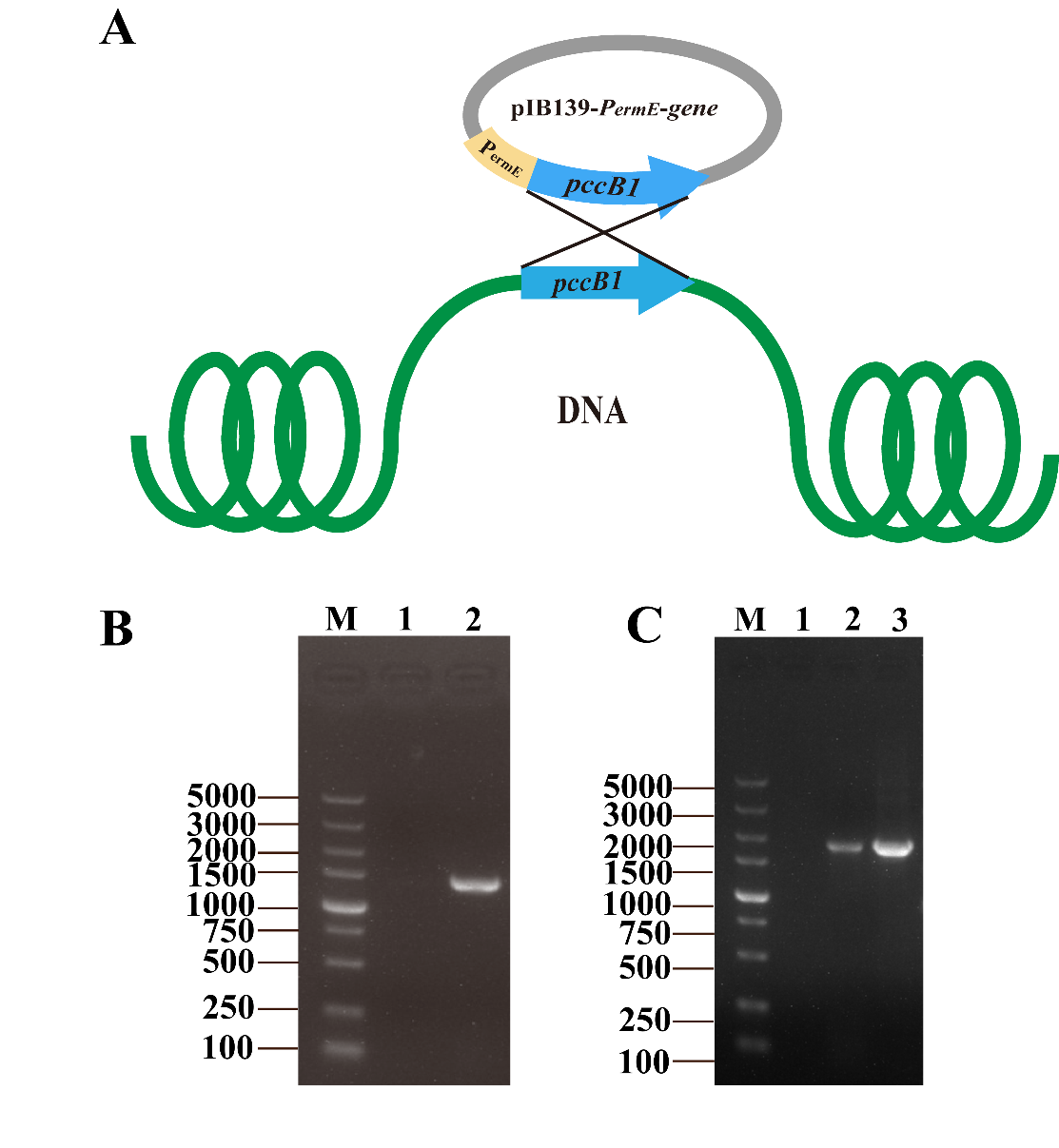


**Figure S1. Construction and Identification of the *pccB1* Gene Overexpression Strain**

A. Construction of the *pccB1* overexpression strain.
B. Amplification of the apramycin resistance (Aprᵣ) fragment. M: DL 5000 DNA Marker; 1: *S. spinosa* genome as template; 2: Transformant genome as template.
C. Amplification of the *P_ermE_-pccB1* fusion fragment. M: DL 5000 DNA Marker; 1: *S. spinosa* genome as template; 2: Transformant genome as template; 3: *pccB1* gene overexpression vector as template.


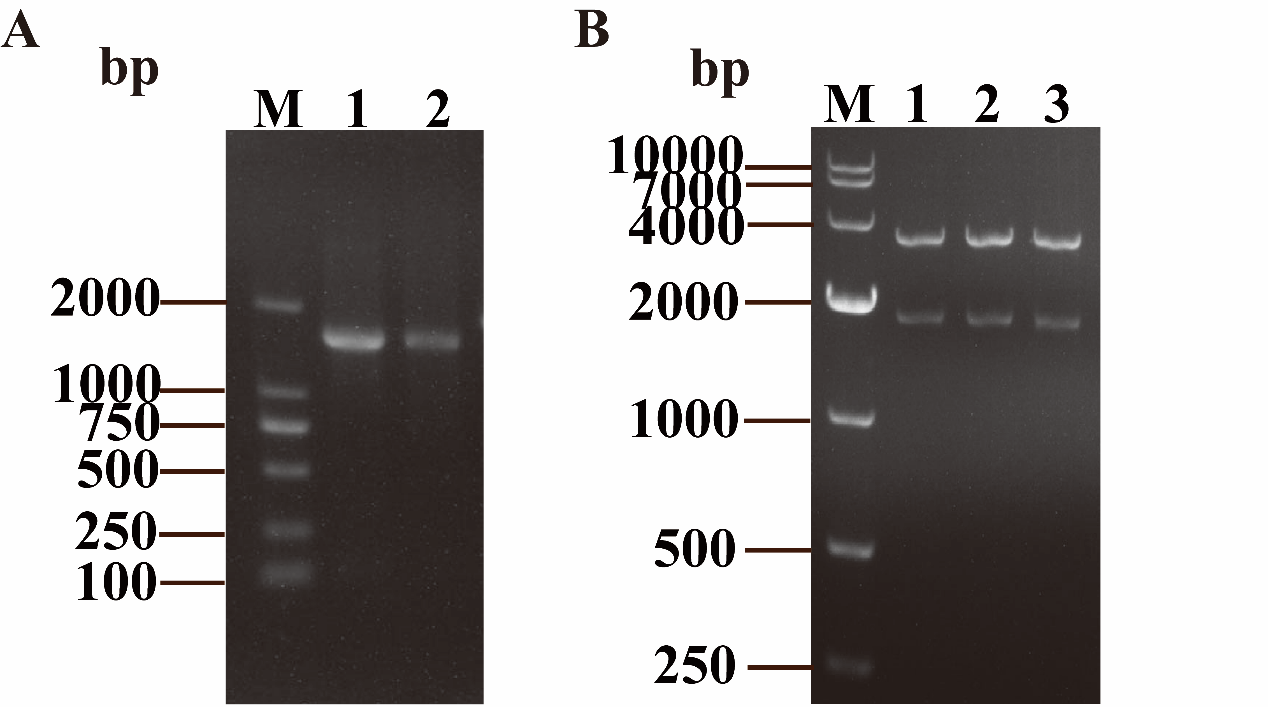


**Figure S2. Construction and Identification of the *pccB1* Gene Overexpression Vector**

A. PCR amplification of the *pccB1* gene. M: DL 2000 DNA Marker; 1–2: Amplification of the *P_ermE_-pccB1* fusion fragment.
B. Double enzyme digestion of the recombinant plasmid. M: DL 10000 DNA Marker; 1–3: Digestion of plasmid pOJ260-*P_ermE_-pccB1* with *Eco*R I/*Hind* III.


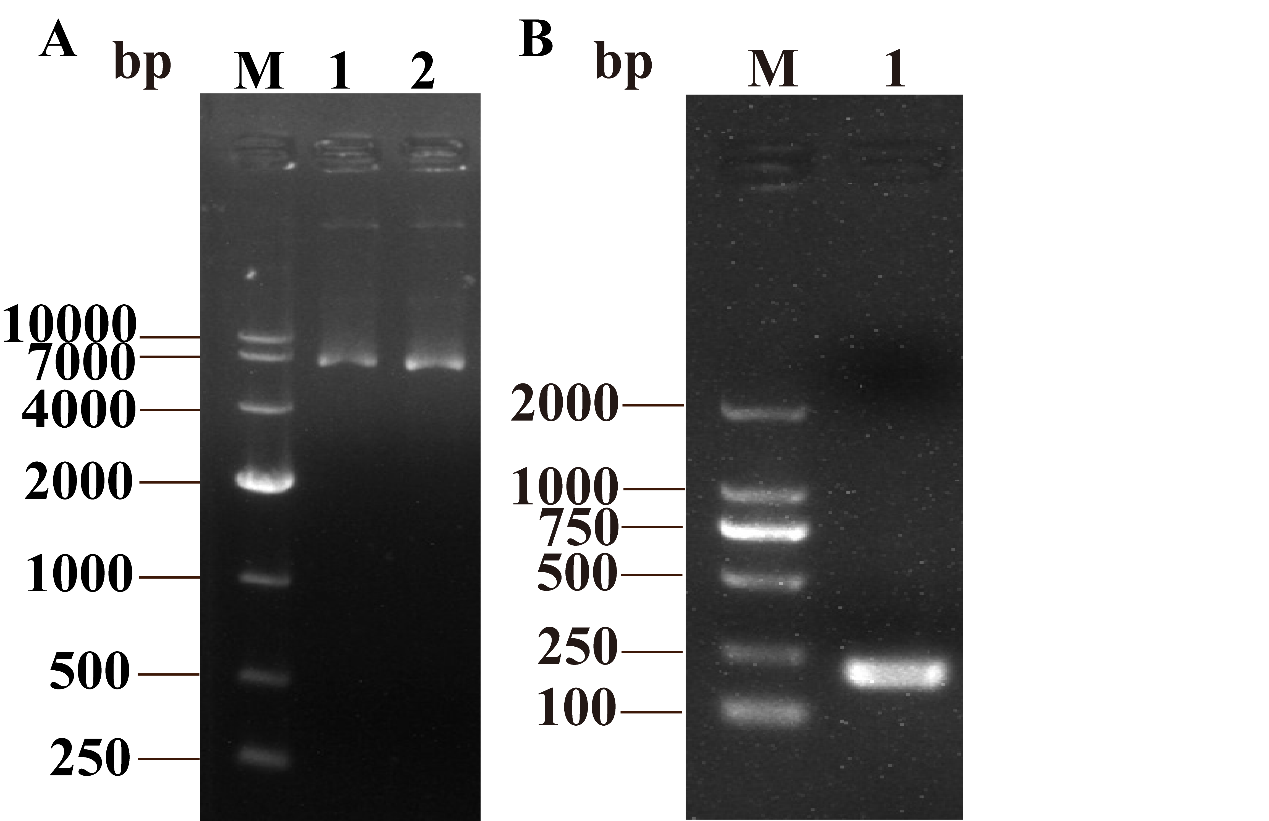


**Figure S3. Construction and Identification of the *pccB1* Gene Repression Vector**

A. DNA bands of the pSET-dCas9 vector. M: DL 10000 DNA Marker; 1–2: Fragments of the pSET-dCas9 plasmid.
B. PCR verification of the sgRNA-*pccB1* fragment in transformants. M: DL 2000 DNA Marker; 1: Fragment of sgRNA-*pccB1*.


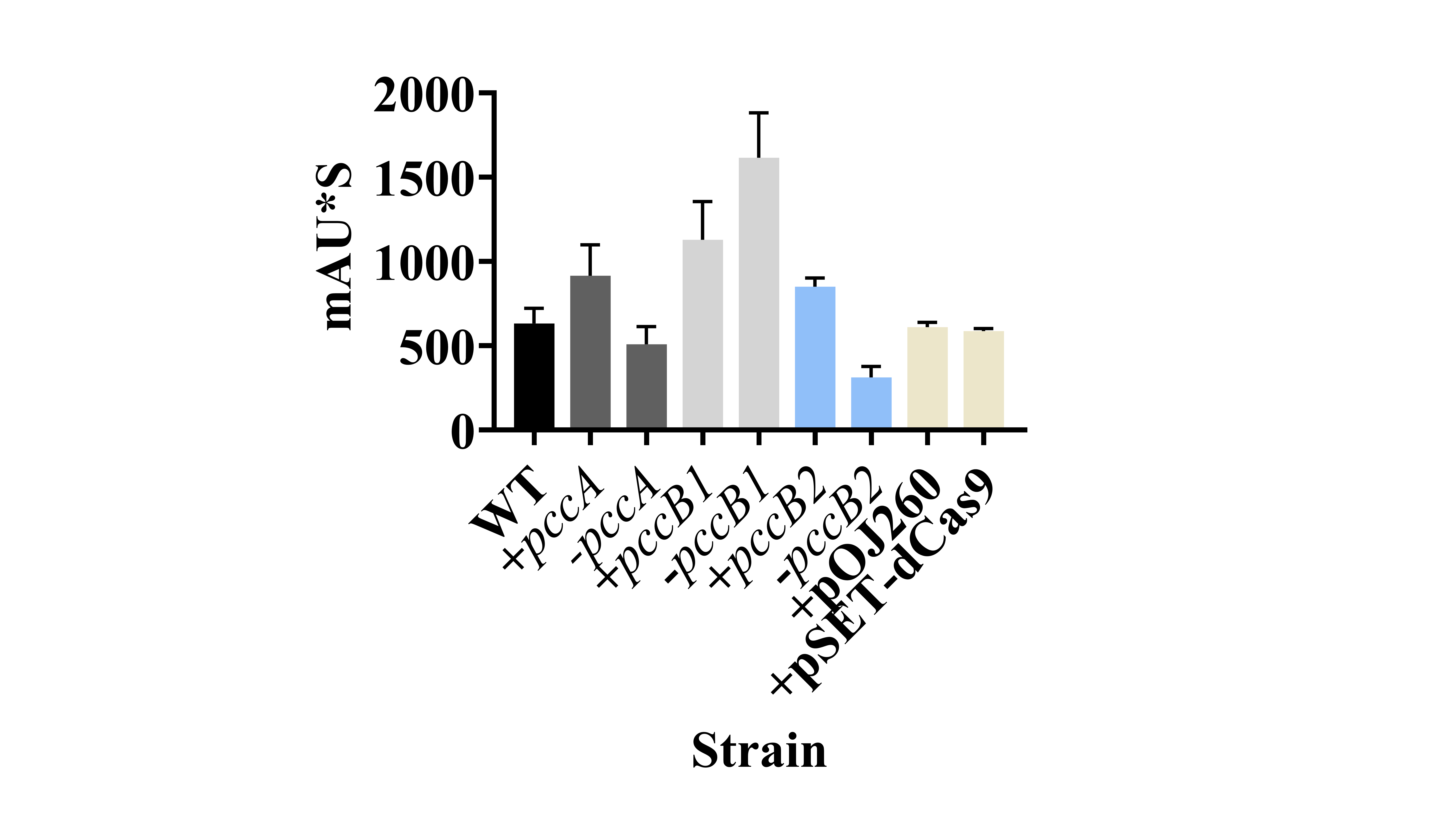


**Figure S4. Determination of spinosad production in WT, WT+plasmid and six engineered strains.**


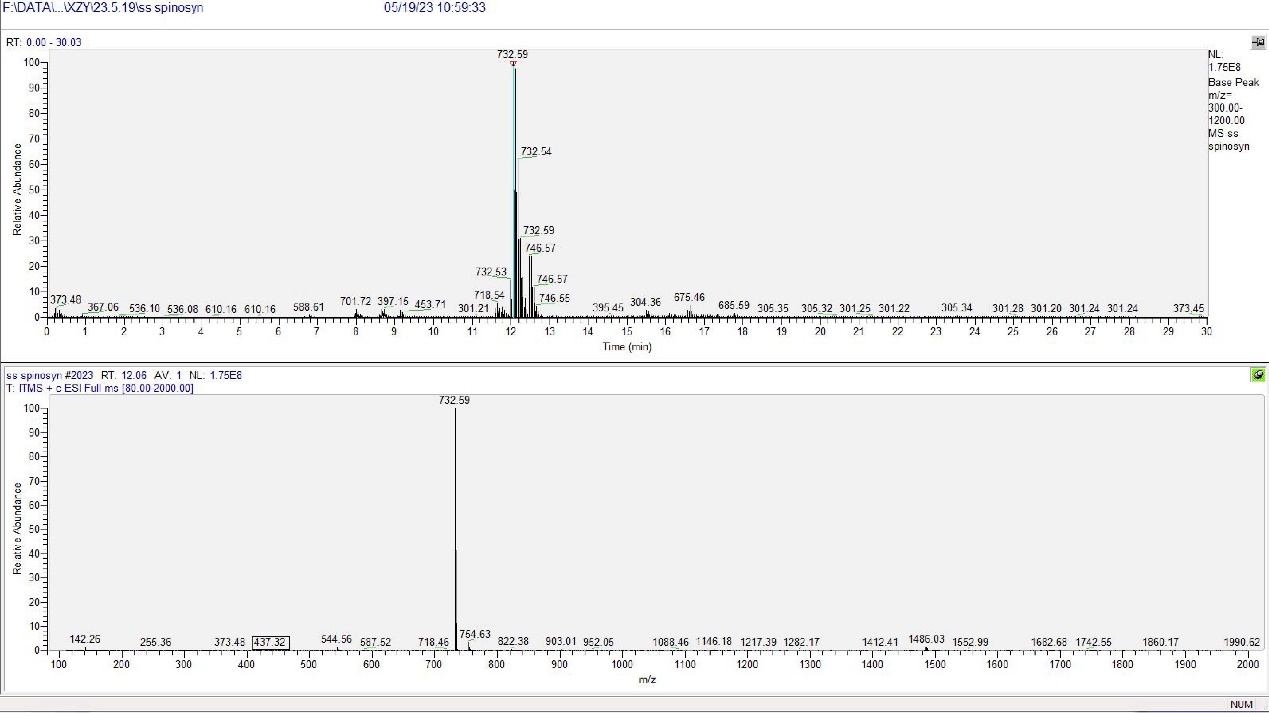
**Figure S5. Identification of Spinosyn by Liquid mass spectrometry identification**

High-resolution mass spectrometry analysis identified characteristic molecular ion peaks corresponding to spinosyn A and spinosyn D at *m/z* 732 [M+H] and 746 [M+H] respectively, exhibiting <1 ppm mass accuracy relative to their theoretical molecular weights. The distinctive isotopic patterns and fragment ions matched authenticated standards, confirming structural integrity of the biosynthesized compounds.


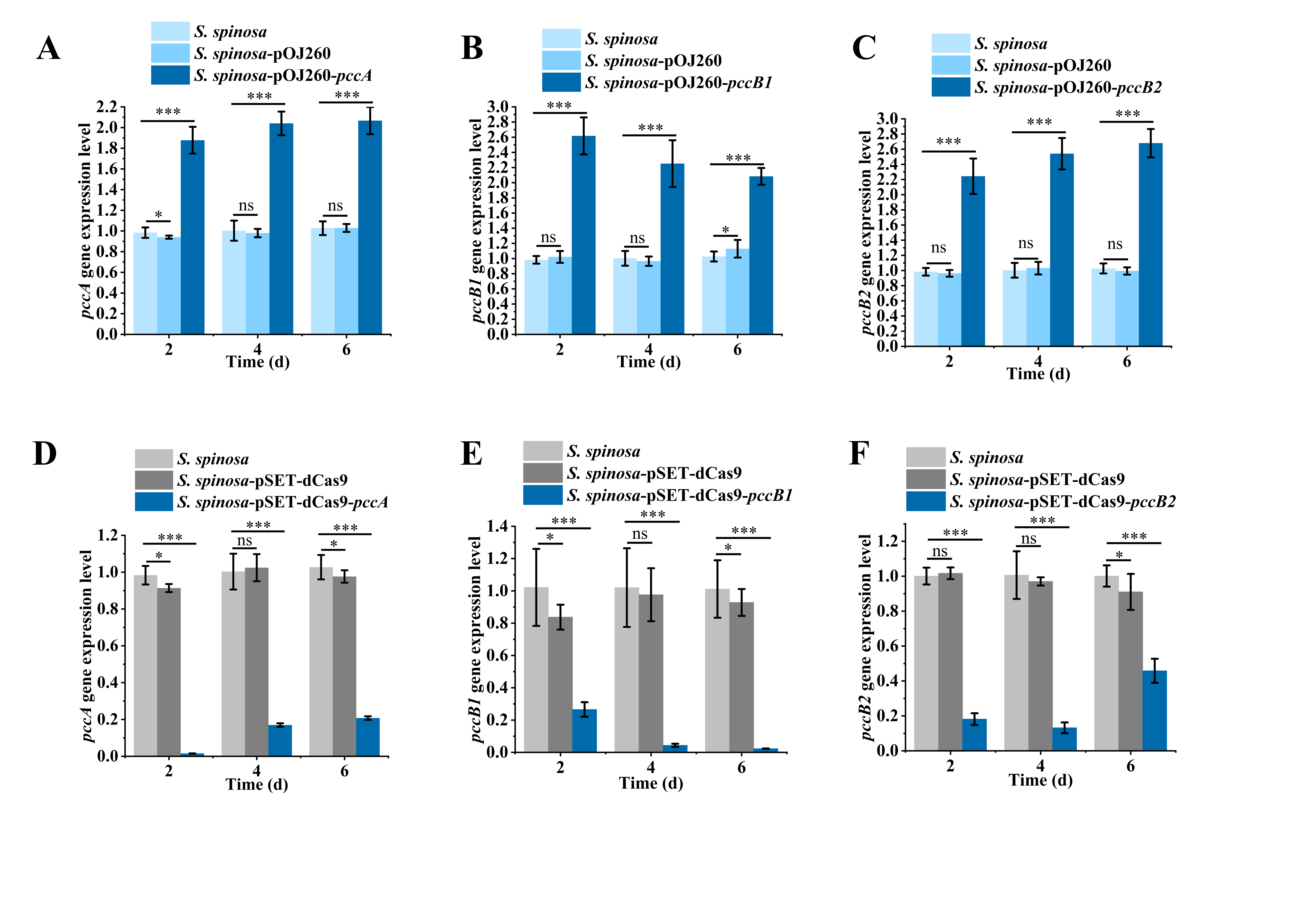


**Figure S6. Transcription analysis of *pccA*, *pccB1* and *pccB2* genes in *S. spinosa*, *S. spinosa*-plasmid and engineered strains**

A, B, C: Transcription level determination of *pccA*, *pccB1* and *pccB2* genes in *S. spinosa*, *S. spinosa*-pOJ260 and overexpression strains. D, E, F: Transcription level determination of *pccA*, *pccB1* and *pccB2* genes in *S. spinosa*, *S. spinosa*-pSET-dCas9 and inhibitory strains.


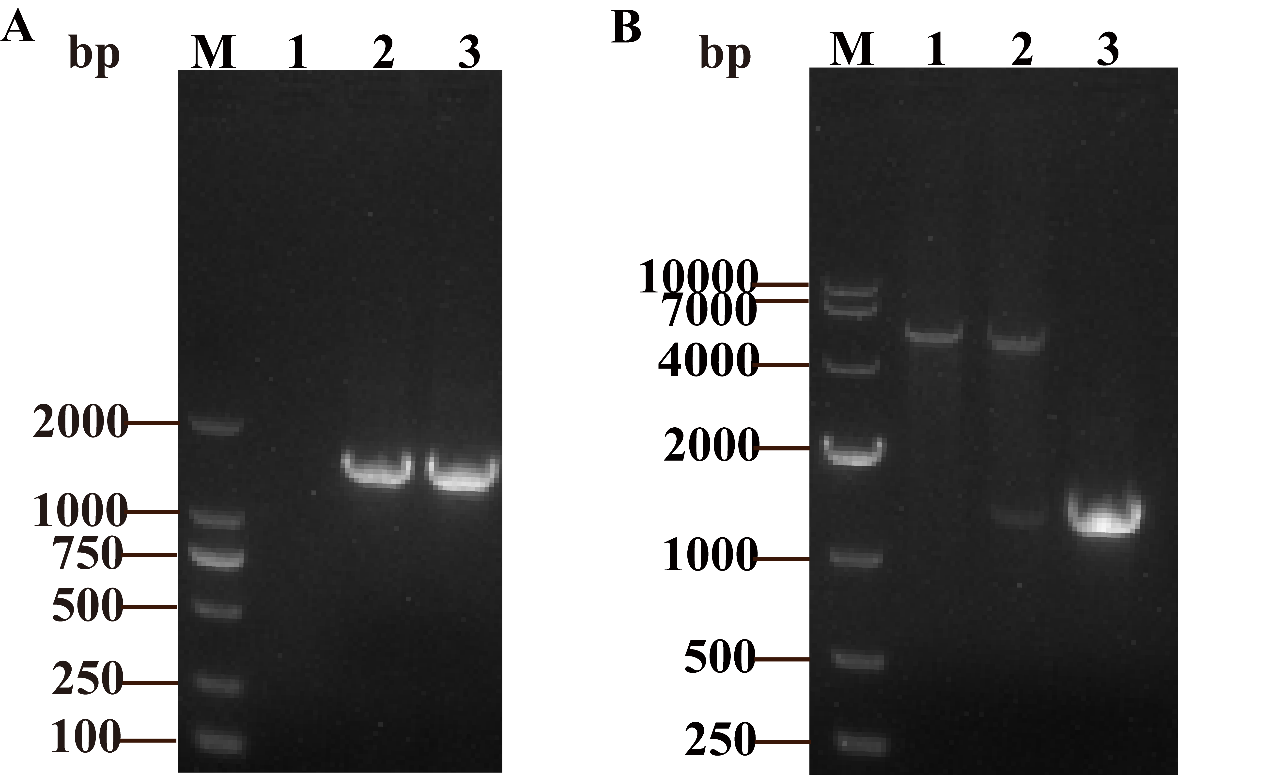


**Figure S7. Construction and Identification of the Heterologous Expression Vector pET28a-*pccB1***

A: PCR verification. M: DL 2000 DNA Marker; 1: pET28a plasmid as template; 2–3: Recombinant plasmid as template.
B: Double enzyme digestion of the recombinant plasmid. M: DL 10000 DNA Marker; 1: *Eco*R I/*Hind* III double digestion of the pET28a plasmid; 2–3: *Eco*R I/*Hind* III double digestion of the recombinant plasmid.


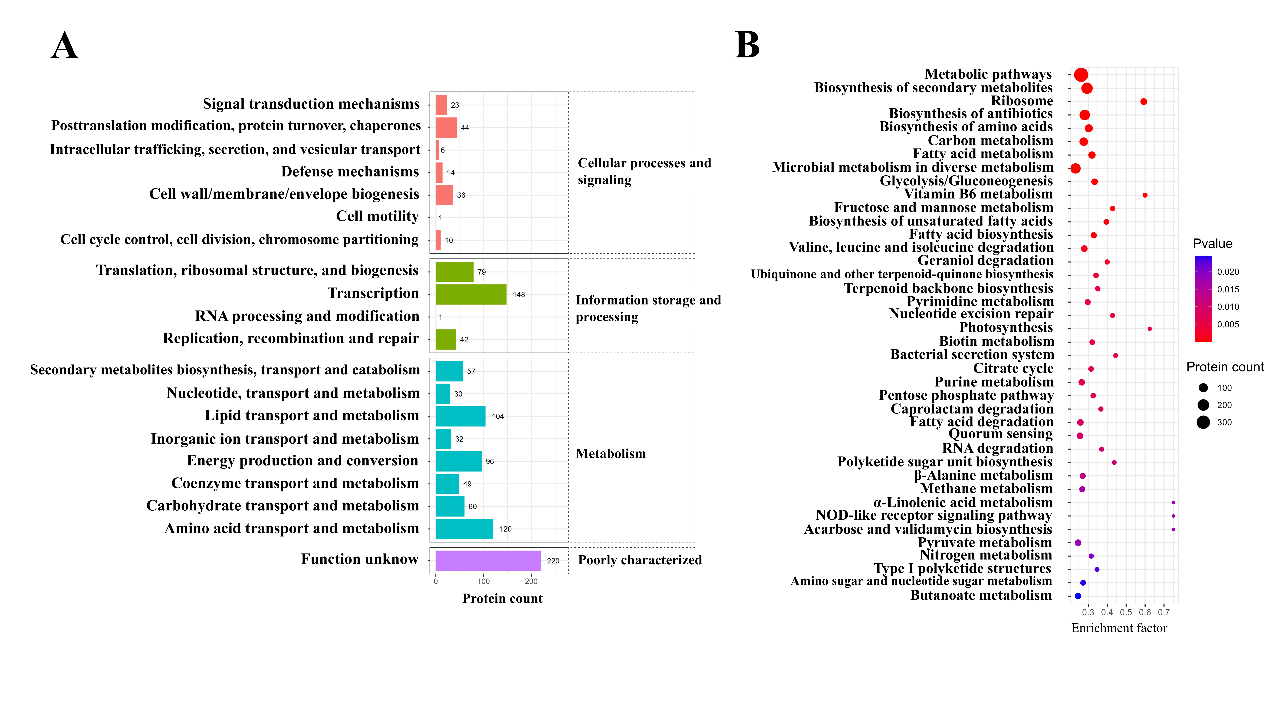


**Figure S8. EggNOG and KEGG Analyses of Differentially Expressed Proteins in WT and *pccB1* Inhibition Strains**

A: EggNOG analysis of upregulated proteins in WT and *pccB1* inhibitory strains.
B: KEGG analysis of upregulated proteins in WT and *pccB1* inhibitory strains.


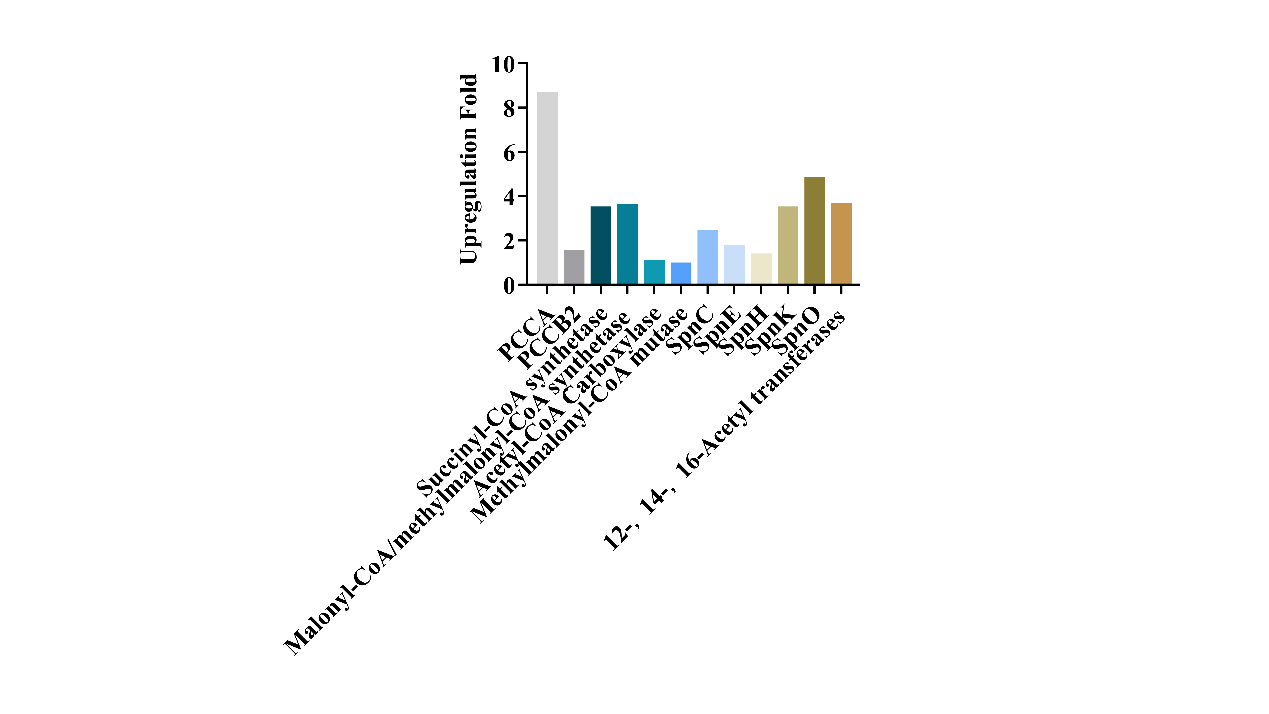


**Figure S9. Fold Changes of Key Upregulated Proteins in the Spinosad Secondary Metabolic Biosynthetic Pathway**


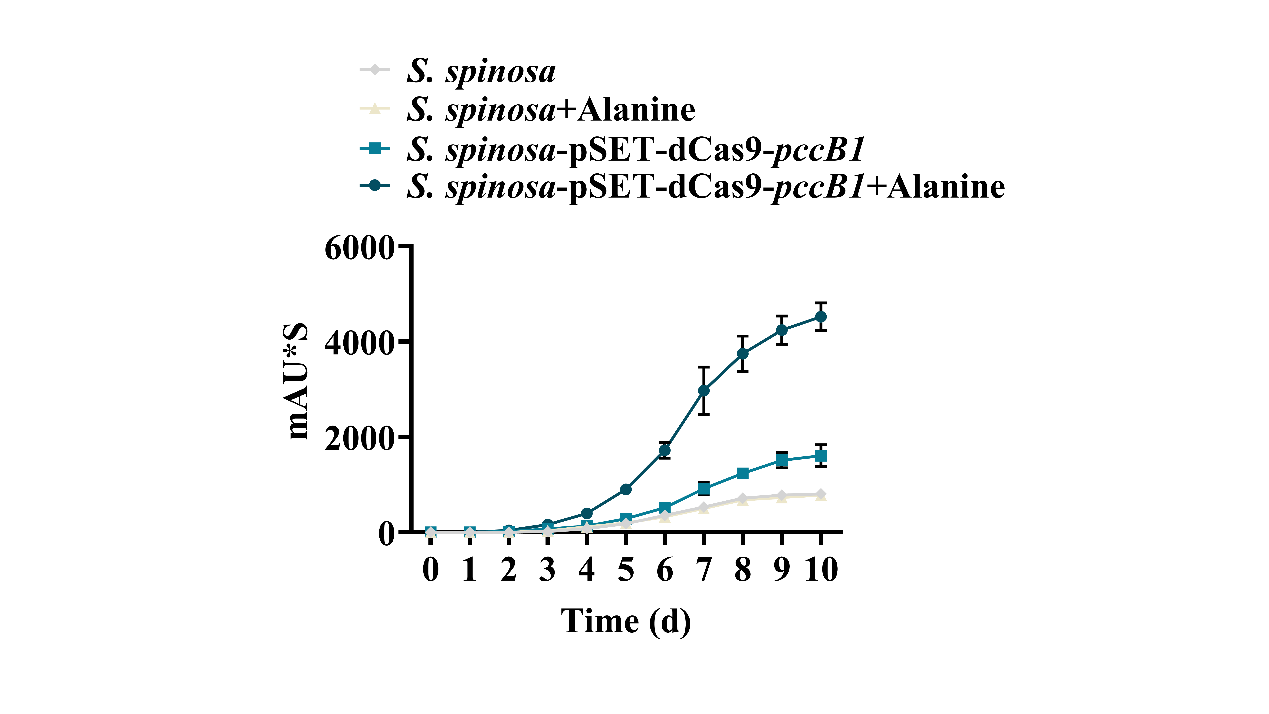


**Figure S10. Accumulation Curve of Spinosad Yield**
